# A short tool to screen self-care preparedness: cross-sectional study in general practice

**DOI:** 10.1093/fampra/cmad107

**Published:** 2023-11-17

**Authors:** Ulla Mikkonen, Nina Tusa, Sanna Sinikallio, Hannu Kautiainen, Pekka Mäntyselkä

**Affiliations:** Institute of Public Health and Clinical Nutrition, University of Eastern Finland, Kuopio, Finland; Wellbeing Services County of North Savo, Health services, Kuopio, Finland; Institute of Public Health and Clinical Nutrition, University of Eastern Finland, Kuopio, Finland; Wellbeing Services County of North Savo, Educational services, Kuopio, Finland; Terveystalo Health Services, Kuopio, Finland; Folkhälsan Research Center, Helsinki, Finland; Institute of Public Health and Clinical Nutrition, University of Eastern Finland, Kuopio, Finland; Clinical Research and Trials Centre, Kuopio University Hospital, Wellbeing Services County Of North Savo, Kuopio, Finland

**Keywords:** chronic disease, cross-sectional studies, patient participation, primary health care, quality of life, self-care

## Abstract

**Background:**

Self-care is crucial in the prevention and treatment of chronic diseases. It is important to identify patients who need support with self-care.

**Objectives:**

This study introduces a self-care preparedness index (SCPI) and examines its associations with health-related quality of life (HRQoL) and other outcomes.

**Methods:**

A cross-sectional study of adults (*n* = 301) with hypertension, coronary artery disease, or diabetes in primary health care. Based on the self-care questionnaire, SCPI was formed. A higher SCPI value indicated better self-care preparedness. We examined correlations and a hypothesis of linearity between SCPI and HRQoL (15D), depressive symptoms (BDI), patient activation (PAM), and health-related outcomes (self-rated health, life satisfaction, physical activity, body mass index [BMI], waist, low-density lipoprotein). Exploratory factor analysis was used to test the construct validity of SCPI.

**Results:**

A total of 293 patients with a mean age of 68 (54.3% women) were included in the analysis. BDI, BMI, and waist had a negative linear trend with SCPI. Self-rated health, physical activity, patient activity, and life satisfaction had a positive linear trend with SCPI. SCPI correlated with HRQoL (*r* = 0.31 [95% CI: 0.20 to 0.41]). Exploratory factor analysis of the SCPI scores revealed 3 factors explaining 82% of the total variance.

**Conclusions:**

SCPI seems to identify individuals with different levels of preparedness in self-care. This provides means for health care providers to individualize the levels of support and counselling. SCPI seems to be a promising tool in primary health care but needs further validation before use in large scale trials or clinical practice.

**Key messages**

- Self-care is essential in the prevention and treatment of most chronic diseases.
- Individuals need different levels of support in self-care.
- Self-care preparedness index identifies different levels of self-care ability.
- Self-care preparedness index has a positive correlation with the quality of life.

## Background

Self-care is crucial in the treatment of most chronic diseases. Health promotion that leads to health behaviour change decreases all-cause mortality and prevents chronic diseases.^1,2^ Effective self-care provides means to influence health behaviours, such as physical inactivity, unhealthy diet, and smoking, and modifiable metabolic risk factors, such as elevated blood pressure, stress, and obesity. These risk factors mediate an increase in the risk of chronic diseases^1^ but also influence the progression of a patient’s existing chronic diseases.^2^ Furthermore, improvements in self-care have a positive impact on the health-related quality of life (HRQoL)^3,4^ and likely reduce health care utilization and costs.^3,5,6^ Since chronic conditions are often managed in primary health care, it is important to study self-care in that setting.

An individualized care plan might support patients in implementing self-care. Many clinical practice guidelines for chronic diseases encourage care planning. However, it does not necessarily bring benefits to such patients whose treatment balance is already good.^7^ To allocate limited healthcare resources appropriately, it would be important to identify the need for support and counselling. One way might be to identify an individual’s self-care preparedness and individualize the levels of support according to that. Specifically, people in a vulnerable position regarding the success of self-care could be identified and supported better.^8^

Self-care can be understood as activities related to medical management (e.g. adherence to diet, medication, and monitoring), role management (e.g. creating and maintaining new meaningful behaviours and life roles), and emotional management (e.g. adaptation to illness in emotional level).^9^ An individual’s capacity, disposition, and action influence each other and lead to achieving successful self-care.^10,11^ Individuals have different capabilities and resources for self-care. Therefore, measuring the patient’s commitment to self-care should be part of the treatment of chronic diseases.^11^ However, there is not generic, easy to use questionnaire for self-care preparedness.

The COM-B model, a theoretical framework for behaviour change illustrates how capability, opportunity, and motivation interact to produce behaviour change.^12^ These 3 factors need to be present for any behaviour to occur. Separate theoretical concepts can be placed within this broader framework. Self-efficacy, patient activation, and health literacy can be understood as individual’s capability whereas inner resources and motivation describe individual’s motivation.^8^ Self-efficacy means an individual’s belief in his or her ability to perform well and to deal with health problems.^13^ Higher self-efficacy is associated with better self-care^14,15^ and also with higher HRQoL and less depression.^16^ Similarly, the concept of patient activation considers an individual’s knowledge, skills, and confidence for managing his or her health and health care.^17,18^ Higher patient activation is associated with better results in clinical outcomes^19,20^ and with higher HRQoL.^20^ Measuring a patient’s activation may help to identify those who need more support in self-care.^20^ However, self-efficacy and patient activation are not enough when considering self-care: health literacy refers to an individual’s skills and environmental conditions that enable them to obtain and apply information to make decisions and take actions that impact their health status^21^ and self-care performance.^22,23^ In addition, health literacy is probably positively associated with HRQOL.^24^ Nevertheless, having chronic conditions and carrying out self-care might be burdensome,^25^ which is why an individual’s inner resources and motivation should be considered. For example individuals with mental health problems are more prone to unhealthy behaviours.^26^

In the present study, we introduce and describe a self-care preparedness index (SCPI) and analyse the relationship of SCPI with HRQoL and other health-related outcomes.

## Methods

### Study design

This was a cross-sectional study of baseline data from the Participatory Patient Care Planning in Primary Care trial (ClinicalTrials.gov ID:NCT02992431). Tusa et al. have published a more detailed description on the study protocol and results at baseline and at a 1-year follow-up.^7,27^ The randomized clinical trial was conducted in Siilinjärvi Health Care Center. Those patients who had their follow-up visit concerning their chronic conditions were recruited between February 2017 and March 2018. The following inclusion criteria were applied: age ≥ 18 years and having diabetes, coronary artery disease or hypertension. We used baseline data from an intervention group (*n* = 301) in the present study. Self-care questionnaires were missing from 6 participants and 2 participants were excluded: one due to being allocated to the wrong group and one due to missing laboratory results. We eventually analysed the data of 293 participants.

### Self-care questionnaire

Participants answered the self-care questionnaire as a part of the participatory patient care planning intervention (Table 1). The development of the self-care questionnaire is described in a project report of implementation of participatory care plans.^28^ In short, the project was aimed at helping the patients’ orientation towards care planning, awaken and motivate them to self-care, consider their own goals and ways to achieve those goals. Originally, the self-care questionnaire was used as a discussion framework between the nurse and the patient and since then, it has been in the clinical use in Finnish primary health care. We quantified the questionnaire items when developing the self-care preparedness index. We assumed that scoring the self-care questionnaire and using it to assess self-care preparedness could also benefit health care professionals.

**Table 1.** The self-care questionnaire that the study participants answered as a part of the participatory care planning intervention.

Which all of the following describe your self-care?	
1. My self-care is good.	2.My self-care needs enhancement.
1 a. I have enough knowledge about my diseases, treatment goals and treatment options.	2 a. I need more information about my diseases and more support with self-care.
1 b. I want to treat myself.	2 b. I want to treat myself, but my inner resources are insufficient.
1 c. I monitor my health and follow my care plan.	2 c. For the most part, my self-monitoring measurements do not meet the treatment goals and I do not know how to improve my self-care.

Table 2 presents the scoring process for SCPI. A higher value given to an answer option indicated higher self-care preparedness. Hence, those answer options that described intention or taking actions towards self-care, knowledge and skills in self-care, or the capacity of inner resources and motivation resulted in higher scores. Patients were able to choose several answer options that they felt described their self-care. This possibility to choose several options gives the opportunity to express ambivalence in self-care preparedness: a person can have preparedness, but also complicating factors. We assume that the ratio of these factors affects preparedness. Preparedness is not only positive or negative but affected by multiple factors. The SCPI ranged from −5 to +5 (Table 2). We used tertiles to define low, moderate, and high self-care preparedness: A total score of −5 to 0 indicated low self-care preparedness; a total score of 1 to 3 moderate self-care preparedness; and a total score of 4 to 5 high self-care preparedness.

**Table 2.** The scoring process of the self-care questionnaire into the SCPI.

	Health literacy ^a^	Patient activation ^b^	Self-efficacy ^c^	Inner resources/motivation ^d^	Score ^e^
1. ***My self-care is good.***					
1 a. I have enough knowledge about my diseases, treatment goals and treatment options.	+	+	+		+2 points
1 b. I want to treat myself.			+	+	+1 points
1 c. I monitor my health and follow my care plan.		+	+	+	+2 points
2. ***My self-care needs enhancement.***					
2 a. I need more information about my diseases and more support with self-care.	-	-			−1 points
2 b. I want to treat myself, but my inner resources are insufficient.		-	-	-	−2 points
2 c. For the most part, my self-monitoring measurements do not meet the treatment goals and I do not know how to improve my self-care.		-	-	-	−2 points

^a^Health literacy: Health literacy refers to an individual’s skills and environmental conditions that enable them to obtain and apply information to make decisions and take actions that impact their health status and self-care performance.^21^

^b^Patient activation: The concept of patient activation considers an individual’s knowledge, skills, and confidence for managing his or her health and health care.^17^

^c^Self-efficacy: Self-efficacy means an individual’s belief in his or her ability to perform well and to deal with health problems.^13^

^d^Inner resources/motivation: An individual’s capacity, motivation and disposition enable self-care.

^e^The total score of SCPI is the sum of the scores on all 6 question options.

### Other outcomes

We measured both patient-reported outcomes and clinical objective outcomes. The detailed description of the following outcome measurements has been reported previously elsewhere.^7,27^ The most relevant patient-reported outcome was HRQoL measured with the 15D.^29^ Other patient-reported outcomes were depressive symptoms measured with the Beck Depression Inventory (BDI)^30^; patient activation measured with the 13-item Patient Activation Measurement scale (PAM)^17^; self-rated health^31^; physical activity (the FIT Index of Kasari)^32^; life satisfaction (a single question about how satisfied the patient was with their life at the point; the answer options ranged from very satisfied, satisfied, somewhat satisfied, unsatisfied, to very unsatisfied); and mental functions (a single question about how the patient felt their mental function compared with their peers; the answer options ranged from very good, fairly good, satisfactory, fairly poor, to very poor).

Three outcome measurements with ordinal categories were re-grouped to allow comparisons between different self-care preparedness groups. In self-rated health, the answer options excellent, very good, and good were pooled as “good.” In life satisfaction, the answer options very satisfied and satisfied were combined as “satisfied.” In self-rated mental health, the answer options very good and fairly good were combined as “good.”

In addition, we asked about alcohol consumption (the first 2 questions of AUDIT-C)^33^ and current smoking habits (yes/no and number of cigarettes per day), additional diseases, experiences of pain, educational background, and relationship status. Clinical outcomes were body mass index (BMI), waist, blood pressure, and low-density lipoprotein (LDL).

### Validity assessment

Based on evidence, improvements in actual self-care behaviours as well as aspects that facilitate self-care (self-efficacy, patient activity, health literacy, inner resources, and motivation) are associated with HRQoL.^3,4,16,20,24^ Thus, we deduced that the validity of SCPI can be indicated by the positive correlation between SCPI and 15D measurement of HRQoL. PAM, which is a validated patient reported outcome measurement, made it possible to evaluate the correlation between a patient’s activity and SCPI. In addition to indicating patient activity, PAM associates with the self-efficacy.^34^ Of our measurements, life satisfaction, self-rated health and depressive symptom can be regarded reflecting inner resources and motivation. Furthermore, the study included measurements for variables reflecting a need for self-care, such as BMI waist, physical activity, and LDL. We explored the construct validity of SCPI with exploratory factor analysis.

### Statistical analysis

The data are presented as means with standard deviations (SD) or counts (*n*) with percentages (%). The hypothesis of linearity was tested using the Cochran–Armitage test for trend, or analysis (ANOVA) with an appropriate contrast. Correlation coefficients between SCPI and HRQoL were calculated with the Spearman method, using Sidak-adjusted (multiplicity) probabilities (significance level) and confidence intervals. Construct validity was studied with an exploratory factor analysis with the iterated principal-factor method for factoring and promax-rotated factor loadings on polychoric correlation matrix was performed to identify related items in the SCPI questionnaire. The strategies used to extract the number of factors were the Kaiser criteria, which determine that components with eigenvalues lower than 1 should be excluded and the screen test of Cattell criteria. The Stata 17.0 (StataCorp LP; College Station, TX, USA) statistical package was used for the analysis.

## Results

Table 3 presents the characteristics of study participants according to tertiles of SCPI. A total of 293 patients with a mean age of 68 years and with 159 (54.3%) women were included in the analysis. Low self-care preparedness was reported by 79 (27.0%) patients, moderate self-care preparedness by 115 (39.2%) patients, and high self-care preparedness by 99 (33.8%) patients. In the sample, the mean (SD) SCPI was 2.22 (2.31). Among women and men, the mean (SD) SCPI was 1.96 (2.35) and 2.51 (2.23), respectively, and the difference between sexes was statistically significant (*P* = 0.041). Differences in age, sex, or education years were not statistically significant between the low, moderate, and high self-care preparedness groups. There were also no differences in chronic conditions, smoking, alcohol use, pain, living alone, and LDL cholesterol.

**Table 3.** The characteristics of patients (*n* = 293) in the baseline according to the self-care preparedness index (SCPI) in primary health care (2017–2018).

	Tertiles of SCPI			*P*-value*
	LOW −5 to 0 *N* = 79	MODERATE 1–3 *N* = 115	HIGH 4–5 *N* = 99	
Women, *n* (%)	50 (63)	61 (53)	48 (48)	0.053
Age, mean (SD)	69 (9)	67 (9)	69 (9)	0.85
Number of education years, mean (SD)	10.0 (3.3)	10.1 (2.9)	10.2 (3.1)	0.59
Living alone, *n* (%)	19 (24)	31 (27)	24 (24)	0.99
Smoking, *n* (%)	8 (10)	16 (14)	10 (10)	0.94
Alcohol consumption**, scale range 0–8	1.7 (1.3)	1.7 (1.5)	1.8 (1.5)	0.62
Physical activity, mean (SD), scale range 0–100	34.4 (21.3)	41.4 (20.0)	45.9 (17.8)	<0.001
Chronic conditions, *n* (%)				
Hypertension	30 (38)	44 (38)	45 (45)	0.48
Coronary artery disease	12 (15)	21 (18)	17 (17)	0.85
Diabetes mellitus	37 (47)	50 (43)	37 (37)	0.42
Additional diseases, *n* (%)				
Musculoskeletal disorders	54 (68)	51 (44)	53 (54)	0.076
Psychiatric disorders	7 (9)	8 (7)	6 (6)	0.48
Pulmonary diseases	8 (10)	18 (16)	13 (13)	0.61
Dementia	2 (3)	2 (2)	0 (0)	0.14
Body mass index, kg/m^2^, mean (SD)	31.0 (6.1)	29.5 (5.5)	27.7 (4.3)	<0.001
Waist circumference (cm), mean (SD)				
Women	102 (16)	99 (16)	95 (12)	0.027
Men	110 (14)	106 (13)	102 (11)	0.003
LDL cholesterol, mmol/l, mean (SD)	2.64 (1.00)	2.75 (0.97)	2.62 (1.01)	0.85
Experiences of pain during the week, *n* (%)	58 (74)	73 (65)	66 (67)	0.36
Rated their self-rated health as good, *n* (%)	26 (35)	64 (57)	69 (70)	<0.001
Rated their mental health as good, *n* (%)	46 (58)	84 (74)	85 (86)	<0.001
Satisfied with life, *n* (%)	50 (66)	85 (75)	84 (87)	0.001
Beck Depression Inventory score, mean (SD), scale range 0–63	7.8 (5.9)	6.5 (5.0)	4.8 (4.5)	<0.001
15D, mean (SD), scale range 0–1	0.830 (0.098)	0.860 (0.100)	0.898 (0.083)	<0.001
Patient activity (PAM), mean (SD), scale range 0–100	65.8 (15.2)	69.1 (14.8)	71.1 (20.1)	0.039

*P-values for linearity, ** Alcohol consumption: the first 2 questions of AUDIT-C.

Depressive symptoms (BDI), BMI, and waist circumference had a statistically significant negative linear trend with SCPI. Likewise, self-rated health, self-rated mental health, physical activity, patient activity, life satisfaction, and HRQoL had a statistically significant positive linear trend with SCPI (Table 3).

**Fig. 1.**
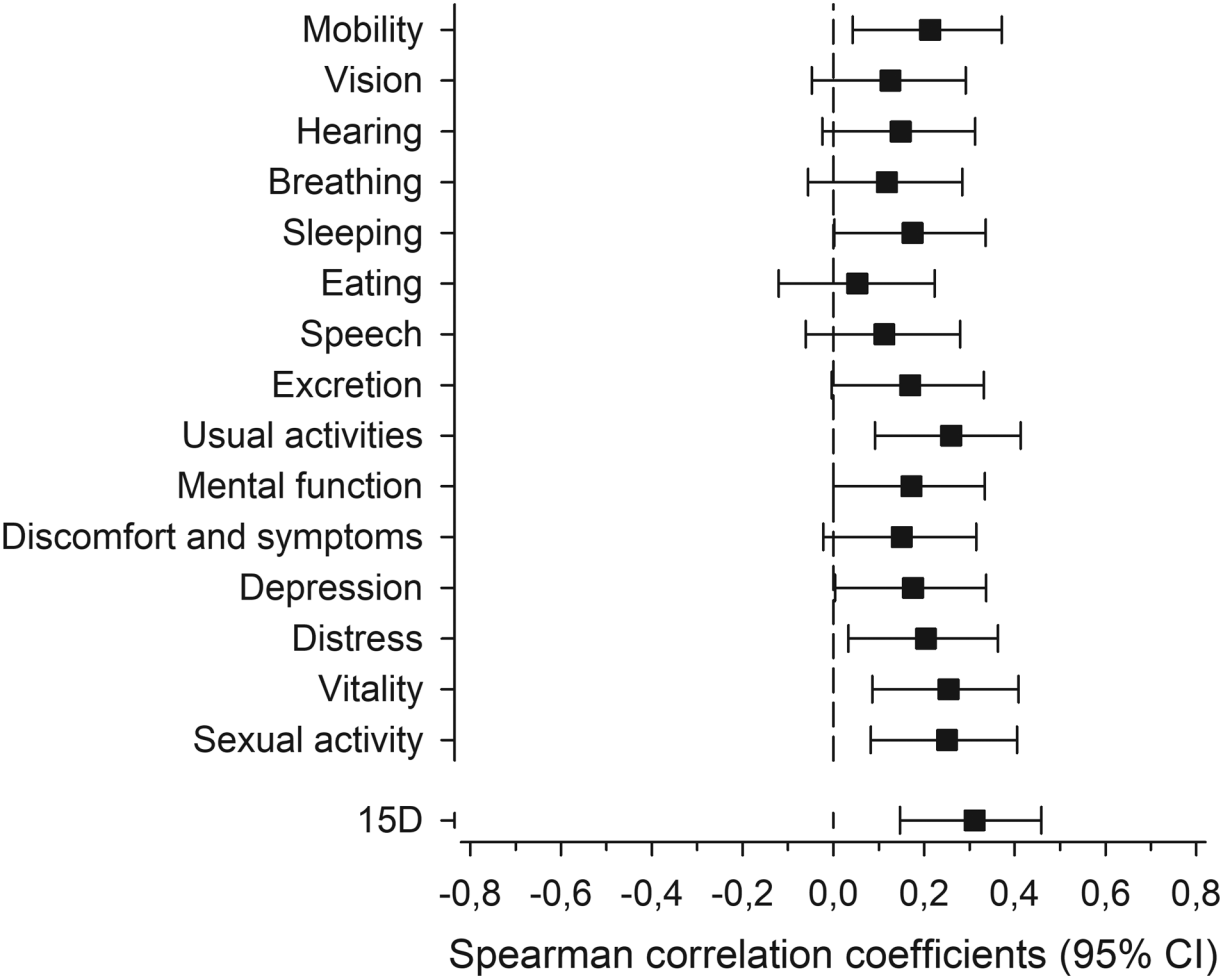
Relationship between the self-care preparedness index and health-related quality of life (15D). Sidak’s adjustment was applied to the correct levels of confidence intervals for multiple testing.

SCPI correlated with HRQoL expressed with the total score of the 15D. Figure 1 shows the correlations between SCPI and the 15D dimensions demonstrating that SCPI correlated with most of the dimensions. The self-care preparedness index also correlated with depressive symptoms (BDI score) (*r* = -0.27 [95% CI: −0.37 to −0.16]) and patient activity (PAM) (*r* = 0.24 [95% CI: 0.12 to 0.34]).

Exploratory factor analysis of the SCPI scores revealed 3 factors. Factor 1 describes patient activation and self-efficacy, factor 2 describes motivation and inner resources, and factor 3 describes health literacy (Table 4). These factors explained 82% of the total variance.

**Table 4.** The exploratory factor analysis with promax-rotated factor loadings of the SCPI-6 items.

	Factor 1	Factor 2	Factor 3
I want to treat myself.	0.89		
I monitor my health and follow my care plan.	0.79		
I want to treat myself, but my inner resources are insufficient.		0.88	
For the most part, my self-monitoring measurements do not meet the treatment goals and I do not know how to improve my self-care.		0.90	
I have enough knowledge about my diseases, treatment goals and treatment options.			0.71
I need more information about my diseases and more support with self-care.			0.96

Coefficients with values <0.50 not shown.

Factors explained 82% of the total variance.

## Discussion

### Summary

SCPI might be useful in identifying patients with varying needs of support and counselling in self-care. It provides a short tool for the patient’s orientation for self-care planning and for health care providers to individualize the levels of support. Compared with patients with a higher SCPI, patients with a lower SCPI were more obese, had lower physical activity, more depressive symptoms, lower self-rated health, lower patient activation, and lower satisfaction with life. Patients with a lower SCPI also experienced the lowest HRQoL. This study generated a hypothesis that this kind of measure could be used as a short generic tool in primary health care.

### Strength and limitations

The strength of the study is novelty. No short screening measure for self-care preparedness is available to the best of our knowledge. Another strength is the study setting, in which the population is a good representative of primary care patients with hypertension, diabetes, or coronary artery disease. Since the study design was implemented in a clinical work setting and participants were recruited from patient flow, the study also produced data from everyday work.

Our study has a few limitations. First, since the study design was cross-sectional, the direction of associations cannot be deduced from these results. Second, the sample size was small and from a local municipality, which limits the generalizability of the results. Third, data were mostly from self-reported questionnaires, which might cause bias due to the patient-reported outcomes. However, bringing out the patient’s experience (patient-reported outcomes; PROMs) might be considered a strength. In addition, we measured clinical outcomes, i.e. objective measurements, which strengthen the reliability of the results. Fourth, detailed information on the development and scientific background of the self-care questionnaire was not available from the original project report.^28^

### Comparison with existing literature

Evidence was the basis for scoring SCPI: self-efficacy,^14,15^ patient activation,^18–20^ health literacy,^22–24^ inner resources, and motivation^8,25^ were identified as important enablers of self-care.^8^ These background theories were also suitable for describing the 3 factors that the exploratory factor analysis revealed suggesting the construct validity of SCPI. The COM-B framework also provides support for this definition.^11,12^ Previous studies have shown that improvements in self-care are related to higher HRQoL.^3,4,35^ SCPI had a positive linear correlation with HRQoL (15D) and patient activation (PAM), suggesting the validity of this measurement. Previously, patient activation has been linked to self-care-related outcomes: in primary health care, multimorbid patients with lower activation were more obese, less physically active and had unhealthy nutrition more frequently than patients with higher activation. Furthermore, patients with lower activation experienced lower HRQOL and self-perceived health.^20^ Similarly, we observed that physical activity, BMI, and waist were associated with SCPI: the higher index value, the better the self-care-related outcomes. Furthermore, patients with higher SCPI reported fewer depressive symptoms and higher life satisfaction, which might be understood as the capacity of inner resources. The relationship between SCPI and self-rated health also strengthens the index’s importance, as self-rated health is a strong predictor of mortality and morbidity.^36^ Likewise, HRQoL predicts mortality,^37^ which is why the index’s linear correlation with HRQoL seems of importance. However, statistically significant differences in the use of alcohol, smoking, or LDL levels were not observed between groups in our study. This might be due to the rather good overall long-term treatment situation related to hypertension, coronary artery disease, and diabetes.

Depression is under-recognized among patients with chronic diseases,^38^ and associated with unhealthy lifestyles,^26^ poor self-management strategies,^35^ chronic conditions^38^ and worse treatment outcomes.^39,40^ Similarly, we observed a negative linear trend with SCPI and depressive symptoms. This underlines the importance of screening depressive symptoms among individuals underperforming in self-care. Depression causes a lack of motivation. Supporting motivation needs special attention among depressed individuals with chronic conditions.

### Implications for research and/or practice

Since chronic conditions are usually treated and followed-up in primary health care, it has a crucial role in promoting self-care. We propose that SCPI might be used as a short screening tool, since it is quite cursory and does not provide specific information for which area of self-care the patient needs support. Hence, the index value acts as a basis for a patient-centred discussion between a patient and health care provider and should lead to an individualized, participatory care plan. However, it is not evident that all primary health care patients benefit from participatory care plans.^7^ From a clinical point of view, there are patients who already perform well in their chronic disease management. Thus, health care can support their self-care with lighter resources. Meanwhile, there are patients who struggle and then fall through the net. These are the patients that health care should identify and support better. The important problem related to chronic disease management generates a question: could it be useful to consider patients’ self-care preparedness and then build a participatory patient care plan for those with lower levels of preparedness? Health care providers should also consider that there are issues concerning health care, such as access to services, continuity of care, care relationship, patients-centredness, and sufficient time and support that facilitate self-care.^23^

SCPI needs further validation before it could be used in large scale trials or clinical practice. SCPI should be compared with already existing disease-specific self-care measurements. Further cohort studies should evaluate the hypothesis that individuals with different levels of preparedness for self-care might be identified with SCPI.

In addition, further trials should examine whether the screening of self-care preparedness would help in targeting health care resources, for example, participatory care plans, better. Longitudinal studies should test whether SCPI at the baseline predicts self-care behaviour (i.e. self-care related outcomes) in follow-up.

## Conclusion

The key role of self-care in the prevention and management of most chronic diseases is evident. We suggest that SCPI might be valuable as a short screening tool to identify patient’s self-care preparedness and to aid patient’s orientation towards self-care planning. Screening self-care preparedness might aid health care providers to individualize the levels of support better. This study generates a hypothesis that SCPI could be used as a short generic tool in primary health care.

## Supplementary Material

cmad107_suppl_Supplementary_Checklist

## Data Availability

The data are available from NT on reasonable request. Due to the protection of individual privacy, the data are not publicly available.
